# In Vitro Characterization of a Nuclear Receptor-like Domain of the Xylanase Regulator 1 from *Trichoderma reesei*

**DOI:** 10.3390/jof8121254

**Published:** 2022-11-27

**Authors:** Thiago M. Mello-de-Sousa, Rita Gorsche, Birgit Jovanović, Robert L. Mach, Astrid R. Mach-Aigner

**Affiliations:** 1 Institute of Chemical, Environmental and Bioscience Engineering, TU Wien, Gumpendorfer Str. 1a, A-1060 Vienna, Austria; 2 Christian Doppler Laboratory for Optimized Expression of Carbohydrate-Active Enzymes, Institute of Chemical, Environmental and Bioscience Engineering, TU Wien, Gumpendorfer Str. 1a, A-1060 Vienna, Austria

**Keywords:** carbohydrate signalling, eukaryotic transactivator, Xylanase regulator 1, circular dichroism, *Trichoderma reesei*

## Abstract

Engineering transcription factors is an interesting research target gaining increasing attention, such as in the case of industrially used organisms. With respect to sustainability, biomass-degrading saprophytic fungi, such as *Trichoderma reesei*, are promising industrial work horses because they exhibit a high secretory capacity of native and heterologously expressed enzymes and compounds. A single-point mutation in the main transactivator of xylanase and cellulase expressions in *T. reesei* Xyr1 led to a strongly deregulated and enhanced xylanase expression. Circular dichroism spectroscopy revealed a change in secondary structure caused by this mutation. According to electrophoretic mobility shift assays and determination of the equilibrium-binding constants, the DNA-binding affinity of the mutated Xyr1 was considerably reduced compared to the wild-type Xyr1. Both techniques were also used to investigate the allosteric response to carbohydrates (D-glucose-6-phosphate, D-xylose, and sophorose) signalling the repression or induction of Xyr1 target genes. The mutated Xyr1 no longer exhibited a conformational change in response to these carbohydrates, indicating that the observed deregulation is not a simple matter of a change in DNA-binding of the transactivator. Altogether, we postulate that the part of Xyr1 where the mutation is located functions as a nuclear receptor-like domain that mediates carbohydrate signals and modulates the Xyr1 transactivating activity.

## 1. Introduction

The filamentous ascomycete, *Trichoderma reesei* (teleomorph *Hypocrea jecorina*, [1]), is a saprophyte that is industrially exploited for its ability to secrete vast amounts of cellulolytic and hemicellulolytic enzymes. Among those are the two main cellobiohydrolases, CBHI and CBHII (EC 3.2.1.91) [2], and the two main endo-β-1,4-xylanases, XYNI and XYNII (EC.3.2.1.8) [3]. Together with other enzymes, they degrade cellulose and xylan to yield oligo- and monosaccharides such as D-glucose, D-xylose, and the transglycosylation product sophorose, among others [4]. These, in turn, affect the expression of these cellulolytic and hemicellulolytic enzymes (reviewed by, e.g., [5,6]).

The Gal4-like Zn_2_Cys_6_ binuclear cluster protein, Xylanase regulator 1 (Xyr1), works as a wide domain activator for the production of all major cellulose- and hemicellulose-degrading enzymes. Accordingly, it was shown that a *xyr1* deletion mutant no longer expresses cellulases or xylanases [7,8]. Furthermore, the transcript formation of the major cellulase-encoding genes, *cbh1* and *cbh2*, encoding CBHI and CBHII, respectively, directly correlates with the transcript pattern of *xyr1*, encoding their activator, Xyr1 [9,10]. In particular, this becomes obvious as both the regulator and these target genes are induced by sophorose and repressed by glucose [10,11]. In contrast, the xylanase-encoding genes *xyn1* and *xyn2* are induced by low D-xylose concentrations and repressed by glucose or high D-xylose concentrations (e.g., [12,13]), and their expression does not precisely correlate with *xyr1* transcript levels. This suggests that the expression of cellulases is strongly dependent on the concentration of available Xyr1, while the expression of xylanases also depends on additional mechanisms [10].

Derntl and co-workers reported on an industrially used *T. reesei* mutant strain, which exhibits a strongly enhanced and de-regulated xylanase-expressing phenotype [10]. Even though carbon catabolite repression, triggered by easily usable carbon sources, is a mechanism affecting xylanase expression in *T. reesei*, this strain was reported to be “glucose-blind”. This strain was obtained from an industrial ancestor strain by UV mutagenesis and subsequent screening for elevated xylanase activity. Genomic analysis revealed that this strain bears—amongst other mutations—a point mutation that results in an A824V transition in the so-called fungal transcription factor regulatory middle homology (FTFRMH)^3^ region of Xyr. The introduction of this mutation into the parental strain and the reconstitution of the wild-type *xyr1* in the mutant strain demonstrated that the xylanase-deregulated phenotype is indeed related to this mutation. Moreover, a detailed analysis (different carbon sources, different time points) of the transcript levels of important Xyr1 target genes (such as *xyn1*, *xyn2*, *cbh1*, *cbh2*) in a strain bearing Xyr1_A824V_ and its parent strain revealed a strong deregulation of endo-xylanase-encoding genes and a strongly elevated, basal level of cellulase encoding genes. A subsequent in silico secondary structure prediction of Xyr1 finds A824 in the middle of an α-helix, which suggests that the A824V mutation might possibly lead to a change in secondary structure, which could be responsible for the observed deregulation of xylanase expression and the generally enhanced cellulase expression [10].

In case of the orthologue of Xyr1 in *Aspergillus niger*, XlnR [14,15], mutants were investigated bearing the following changes: the mutation V756F and the introduction of stop codons after residues L668 and G797. All modifications resulted in higher xylanolytic activity of the fungus as well as an increased expression of target genes of XlnR, such as *xlnB* (coding for endoxylanase B), even in repressing conditions using D-glucose as the sole carbon source [16]. The region that contains these mutations is located between a putative coiled-coil region and a C-terminal transcriptional activation domain, and is probably related to the modulation of activity of XlnR by D-glucose [16].

Circular dichroism (CD) spectroscopy is very useful for the characterisation of the protein secondary structure (in % α-helix, β-sheets, turns), as well as structural changes resulting from DNA–protein, protein–protein or protein–ligand interactions (e.g., reviewed by [17,18]). Especially in the case of transcription factors, this method is used to investigate the influence of DNA-binding on the protein and DNA structure, an example of which is the investigation into structural changes of the transcription factor NF-κB induced by DNA binding [19].

Though in an earlier study on *T. reesei* an unexpected phenotype was observed and could be related to a A824V mutation in Xyr1, the molecular mechanism remained unsolved. In this study, we used CD spectroscopy, blue native polyacrylamide gel electrophoresis (BN-PAGE), and electrophoretic mobility shift assay (EMSA) to investigate the influence of the previously described mutation on the secondary structure of Xyr1 and its DNA-binding affinity. Finally, the response of the two Xyr1 variants to carbohydrates in the form of conformational changes mediating different signals, which affect (hemi)cellulase expression (D-glucose-6-phosphate, D-xylose, and sophorose), was investigated concerning the putative function of this part of Xyr1 as a nuclear receptor (NR)-like domain.

## 2. Materials and Methods

### 2.1. Plasmid Construction

A 2945 bp-fragment comprising the T7 promoter, the *lac* operator, and the coding region of *xyr1* fused with a C-terminal six-histidine tag was chemically synthesized (GeneArt^®^, part of Life Technologies, Paisley, UK) with codon optimization for *Escherichia coli*. This expression cassette was cloned into the Novagen^®^ pET28a vector (Merck, Darmstadt, Germany) using *Bgl*II/*Not*I restriction sites and yielding pTS1. Likewise, the mutated gene version, *xyr1_A824V_*, bearing the A824V transition, was chemically synthesized and cloned into pET28a, yielding pTS2. Insertion of the correct fragments into both expression vectors was confirmed by restriction profile analysis and automated sequencing (LGC Genomics, Berlin, Germany).

### 2.2. Protein Expression and Purification

A total of 300 mL of LB medium with D-glucose (1% *w*/*v*) and kanamycin (50 µg/mL) was inoculated with *E. coli* BL21(DE3)pLysS (Promega, Madison, WI, USA), carrying the respective expression vectors. At OD_600_ protein expression was induced by adding IPTG to a final concentration of 0.5 mM. The culture was incubated at 18 °C for 24 h. The cells were harvested by centrifugation and stored frozen at −20 °C overnight. Cells were then resuspended in a 10 mL binding buffer (0.5 M NaCl, 20 mM Tris-HCl, 5 mM imidazole, pH 7.9) and sonicated using a Sonifier^®^ 250 Cell Disruptor (Branson, Danbury, CT, USA) (power 40%, duty cycle 70%, power for 30 s, pause for 30 s, 4 cycles). After centrifugation, the protein (105 kDa) was purified from the extract using Novagen^®^ HisBind^®^ resin (Merck) and a modified elution buffer (0.5 M NaCl, 20 mM Tris-HCl, 120 mM imidazole, pH 7.9), according to the manufacturer’s guidelines.

### 2.3. Circular Dichroism Spectroscopy

Before CD spectroscopy measurements, PD-10 columns (GE Healthcare, Uppsala, Sweden) were used for exchanging buffer to buffer A (50 mM Tris, 200 mM NaCl, 50 mM NaH_2_PO_4_, 10% (*v*/*v*) glycerol, pH 7.5). The protein samples were centrifuged at 14,000× *g* for 10 min to remove aggregates, and thereafter, the protein concentration was determined using Bio-Rad Protein Assay (Bio-Rad, Hercules, CA, USA). A total of 200 µL of a 250 nM protein solution was used for CD spectroscopy measurements. To study DNA binding to the URR of *xyn1*, synthetic complementary oligonucleotides (Sigma-Aldrich, St. Louis, MO, USA) were annealed and used at a final concentration of 75, 150, 225, 300, 375, 450, 525, 600, 675, 750, 825, and 900 nM. Oligonucleotide sequences are provided in Table 1. To study the influence of D-glucose-6-phosphate, D-xylose, and sophorose, the sugars were used at final concentrations (or sugar-to-protein ratios, respectively) of 40 (1:5), 200 (1:1), and 1000 nM (5:1). Measurements were carried out in 0.2 cm SUPRASIL^®^ quartz cells (HellmaAnalytics, Müllheim, Germany) in a J-815 CD Spectrometer (Jasco, Tokio, Japan) at 22 °C. CD spectra of the proteins were collected from 260–200 nm as an average of 3 scans and subtracted baseline in order to exclude buffer influences. Data are presented as the mean residue ellipticity [θ] in deg cm^2^ dmol^−1^, i.e., (millidegrees × MRW)/(pathlength in mm × concentration in mg/mL), where MRW (mean residue weight) is 109.69 Da. The equilibrium-binding constants (Kd) of the DNA–protein complexes were determined by following the changes of [θ]_222_, using the non-linear least squares method to fit a curve based on Engel’s equation [20] with n = 2 to the measured data. For an investigation of the temperature-dependent unfolding, the temperature was increased from 10 °C to 100 °C at a rate of 5 °C/min for the heat-denaturation process. Both proteins were used in a concentration of 250 nM in buffer A.

### 2.4. Electrophoretic Mobility Shift Assay

Synthetic FAM-labelled oligonucleotides (Sigma-Aldrich) used for EMSA were annealed with their complementary oligonucleotides (Table 1). The protein–DNA-binding assay and non-denaturing polyacrylamide gel electrophoresis were performed essentially as described previously [21]. Binding was achieved by incubating 350 ng of the heterologously expressed Xyr1 or Xyr1_A824V_ with 15 ng of a labelled, double-stranded DNA fragment in buffer A (10 min, 22 °C). The addition of p(dIdC) at a final concentration of 0.2 µg/µL and an unspecific oligonucleotide (CKT067/CKT068) avoid unspecific binding. Fluorescence and image analysis of the gels was carried out using a Typhoon 8600 variable mode imager (Amersham Bioscience, part of GE Healthcare, Chicago, IL, USA).

### 2.5. Blue Native Polyacrylamide Gel Electrophoresis

The separation of the protein complexes was performed with 200 nM of each protein in non-denaturing conditions according to the modified protocol [22]. For the second dimension, proteins were denatured in the gel strip after separation by BN-PAGE and subsequently subjected to SDS-PAGE, as previously described [23]. The identity of the proteins was confirmed by Western blot.

## 3. Results

### 3.1. A Single-Point Mutation in the Zinc Finger Regulatory Protein Xyr1 Changes Its Secondary Structure

**Figure 1:**
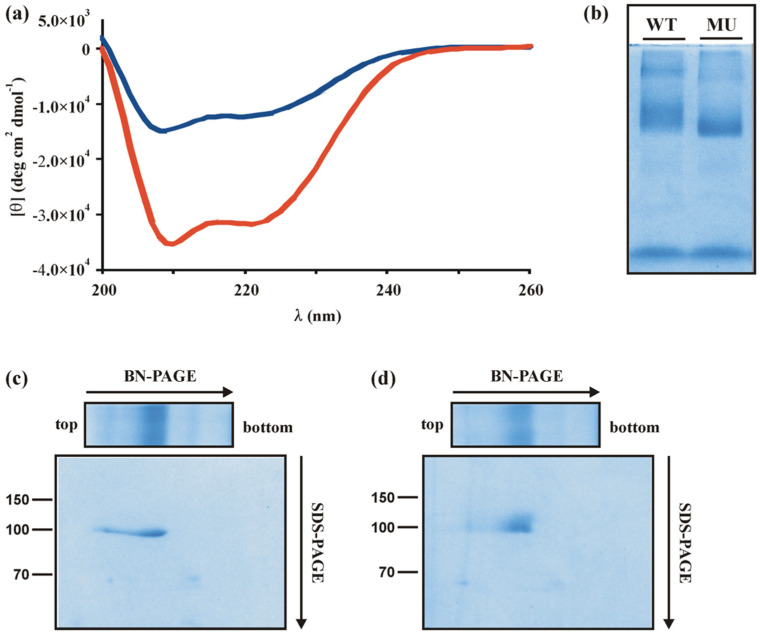
Characterization of the wild-type Xyr1 and the A824V mutant protein. (**a**) Far-UV spectra (200–260 nm) from CD analyses of Xyr1 (red line) and Xyr1_A824V_ (blue line) at 22 °C are given. (**b**) Blue native polyacrylamide gel electrophoresis (BN-PAGE) of the wild-type Xyr1 (WT) and the mutated Xyr1 (MU). Subsequent SDS-PAGE of the wild-type Xyr1 BN-PAGE lane (**c**) and the Xyr1_A824V_ BN-PAGE lane (**d**). Size is indicated in kDa.

Recently, it was demonstrated that a mutation in the main transactivator Xyr1, namely a A824V transition, leads to a glucose blind, (hemi)cellulase-expressing phenotype [10]. Generally, transcript formation in the mutant strain seems to be carbon source-independent or non-responsive. As Derntl and co-workers reported that the mutation is located in the α-helices-rich, C-terminal part of the domain that might lead to structural changes of the protein, we investigated this assumption by employing CD spectroscopy. Therefore, the wild-type Xyr1 and the Xyr1_A824V_ were heterologously expressed in *E. coli*. The spectrum of the negative ellipticity strongly differed between the wild-type Xyr1 and the Xyr1_A824V_ (Figure 1a), thus, demonstrating that these proteins vary in their secondary structure. The mutation A824V resulted in a 61% decrease in mean residue ellipticity at 222 nm and a strong decrease in the fraction of residues involved in helical conformation, hence, indicating a possible role of this region for a specific folding of Xyr1. A BN-PAGE of both Xyr1 variants revealed that both of them are present as monomers (strong bands in the middle of the BN-PAGE) and homodimers (fainter, upper bands in the BN-PAGE) in comparable ratios (Figure 1b). A subsequent SDS-PAGE provided evidence that all bands are different agglomerations of the Xyr1 protein (104 kDa) (Figure 1c,d). Therefore, a different protein–protein interaction of the two variants is not responsible for the different CD spectra. Moreover, the observation that both monomer and dimer migration differed between Xyr1 and Xyr1_A824V_ (Figure 1b) supports the assumption of a structural change caused by the point mutation.

### 3.2. The A824V Mutation Changes DNA-Binding Properties of Xyr1

**Figure 2:**
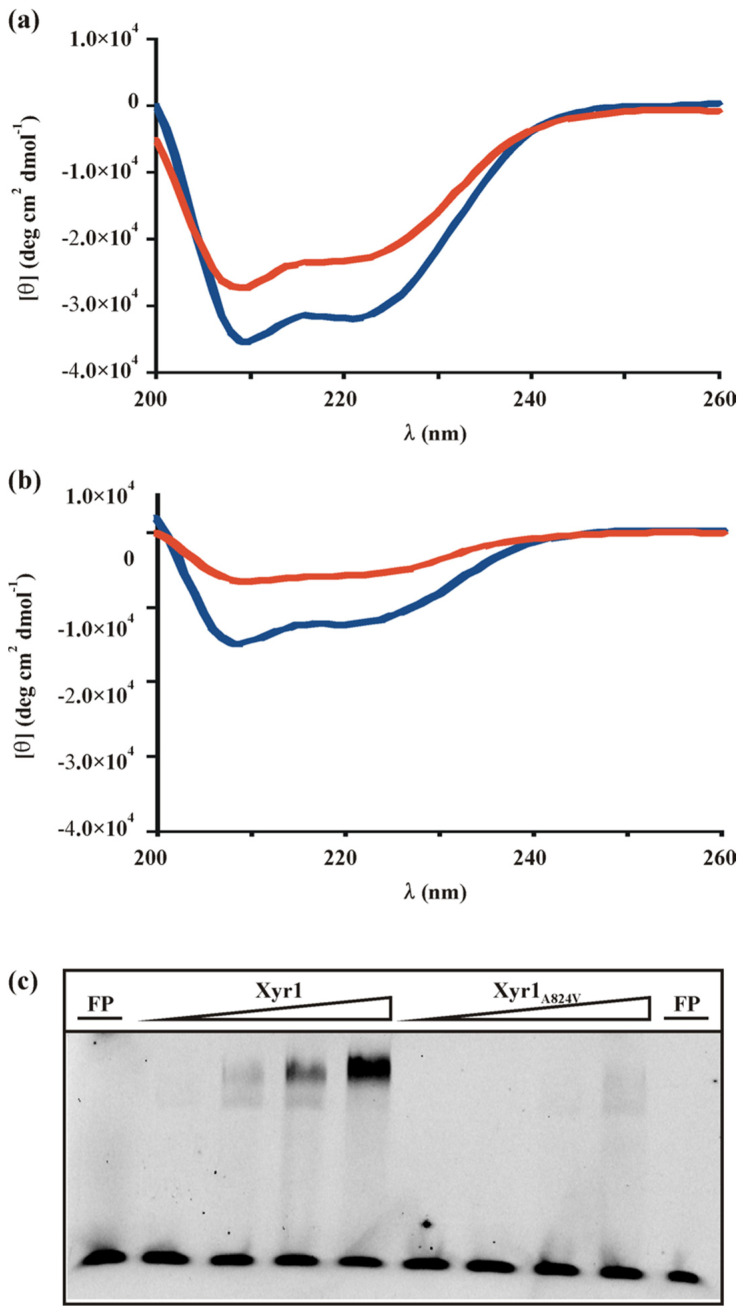
Analyses of Xyr1 and Xyr1_A824V_ binding to target DNA. Far-UV spectra (200–260 nm) of a CD spectroscopy analysis of Xyr1 (**a**) or Xyr1_A824V_ (**b**) alone (blue lines) and their respective protein–DNA complexes (red lines) using a *xyn1* URR fragment (−430 to −396 bp from ATG) in a molar ratio of 1:1. Data were plotted after correction for the contribution of the DNA. (**c**) EMSA analysis of Xyr1 and Xyr1_A824V_ DNA-binding behaviour using increasing amounts of protein (170 ng, 340 ng, 680 ng, and 1360 ng, respectively) and a fluorescently labelled *xyn1* URR fragment. FP, free probe.

To investigate whether the change in secondary structure of Xyr1 caused by the A824V mutation also influences its DNA-binding properties, we analysed Xyr1 binding to the upstream regulatory region (URR) of the *xyn1* gene, the expression of which was affected by the mutation and which is a well-studied target gene within the Xyr1 regulon. For this purpose, CD spectroscopy analysis was used again. In the presence of the double-stranded DNA probe, we found indication for protein–DNA interactions for both proteins, reflected by changes in the spectra of the protein alone compared to protein with target DNA. These changes indicate a change in secondary structure content (Figure 2a,b). The observed reduction in the negative ellipticity due to a potential DNA interaction resulted in a 27% loss of α-helical content for the wild-type Xyr1 and a 53% loss for the Xyr1_A824V_. Here, again, we applied BN-PAGE as a control experiment and found that the presence of DNA did not affect the monomer-to-homodimer ratio of both Xyr1 variants, and thus, is not responsible for the differences in CD spectra (see Supplementary Data, Figure S1a).

As CD spectroscopy analyses are not a direct proof for a protein–DNA complex formation, we performed EMSA analysis employing the same fragment of the URR of *xyn1* as a fluorescently labelled probe. Using different amounts of wild-type Xyr1, two shifts in mobility could be observed at lower concentrations of the protein (Figure 2c). This observation is in accordance with previous findings that reported the binding of Xyr1 to the *xyn1* URR as a monomer and as a dimer [24], yielding two shifts in EMSA experiments. The application of increasing amounts of protein favours binding as a dimer in a concentration-dependent way (Figure 2c, compare [24]). Using Xyr1_A824V_ yielded only a weak double shift (monomer and dimer) at the highest protein concentration (Figure 2c), indicating a decrease in the protein–DNA complex formation due to the A824V mutation.

### 3.3. Determination of the Equilibrium Dissociation Constant of the Xyr1 Variants

**Figure 3:**
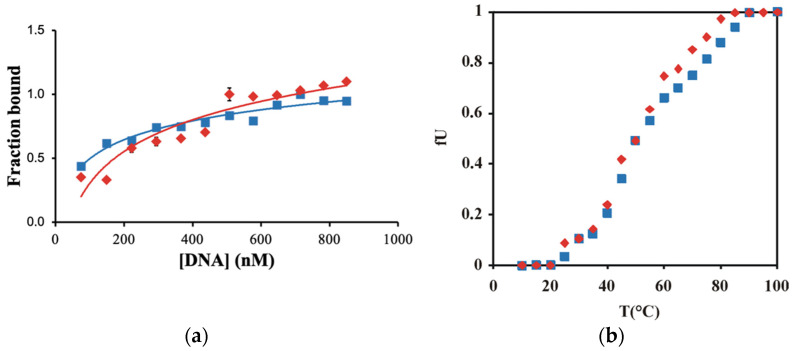
CD spectroscopy-based analysis of the Xyr1 variants. (**a**) Determination of the equilibrium dissociation constants (Kd). Titrations were performed with Xyr1 (blue squares) or Xyr1_A824V_ (red triangles) and increasing amounts of DNA (*xyn1* URR fragment, -430 to -396 bp from ATG), ranging from 0 to 900 nM. The values are means of three independent experiments. Error bars indicate standard errors of the means. If no error bar was depicted, standard deviation was below 5%. (**b**) Temperature-dependent unfolding of Xyr1 and Xyr1_A824V_. The apparent fraction of unfolding (fU) of the proteins Xyr1 (blue squares) and Xyr1_A824V_ (red triangles) was obtained by (θ_N_ − θ)/(θ_N_ − θ_U_), where θ_N_ and θ_U_ are the values of the native and the unfolding state, respectively, and θ is the observed value of θ_222_ at a given temperature. The value of fU was plotted as a function of the temperature.

At first glance, the decreased ability of Xyr1_A824V_ to form complexes with *xyn1* URR contradicts the superior xylanase formation observed in the *T. reesei* strain bearing this mutation. Therefore, the equilibrium dissociation constants of both wild-type Xyr1 and Xyr1_A824V_ were determined. For this purpose, a constant amount of protein with varying amounts of target DNA was analysed by CD spectroscopy following the changes of the mean residue ellipticity at 222 nm. Then, a non-linear least squares method was used to fit a curve based on Engel’s equation [20] to the measured data. Figure 3a depicts the curve fitting results of both Xyr1 variants. The higher Kd value of Xyr1_A824V_ (523 nM) in comparison to the one of the wild-type Xyr1 (128 nM) indicates a lower DNA-binding affinity for Xyr1_A824V_ and supports the former findings of this study.

As this is a rather unexpected finding in the case of a transactivator’s mutation, which caused an enhanced deregulated xylanase production in the respective strain, we assume that the regulatory mechanism is not a simple matter of change in Xyr1–DNA affinity. The reason can neither be found in a general change in protein stability as the temperature-dependent unfolding of both proteins demonstrated that the point mutation had hardly any effect on the stability of the protein (Figure 3b).

### 3.4. Conformational Response to Carbohydrates Is Partly Lost in Xyr1_A824V_

**Figure 4:**
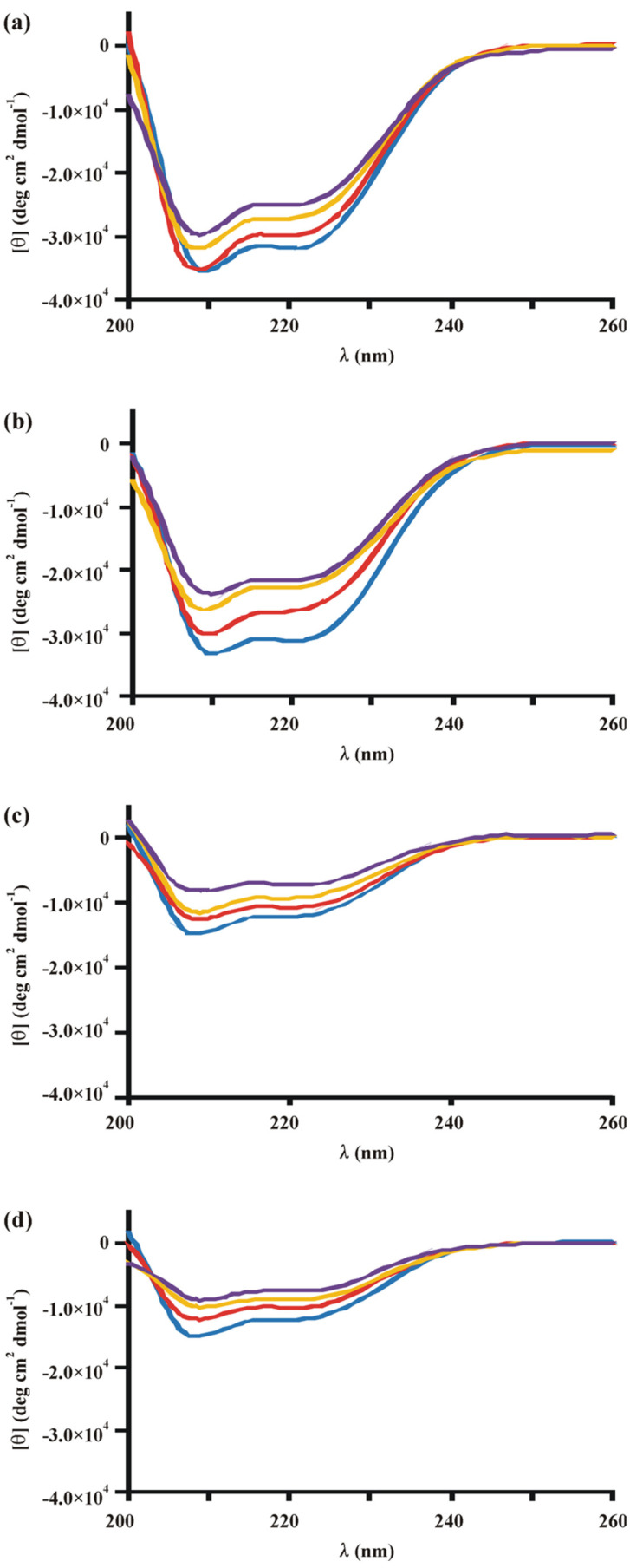
CD analyses of Xyr1 and Xyr1_A824V_ in the presence of carbohydrates. Far-UV spectra (200–260 nm) of Xyr1 (**a**,**b**) or Xyr1_A824V_ (**c**,**d**) in the presence of D-glucose-6-phosphate (**a**,**c**) or D-xylose (**b**,**d**) applying molar ratios (protein to carbohydrate) of 1:0 (blue lines), 5:1 (red lines), 1:1 (yellow lines), and 1:5 (purple lines). Data were plotted after correction for the contribution of the carbohydrates.

As it is known that in eukaryotes the presence of a certain metabolic signal can cause conformational changes in a regulatory protein, this prompted us to investigate possible allosteric modulations of Xyr1 induced by certain carbohydrates. Therefore, we analysed both proteins alone and in the presence of D-glucose-6-phosphate (the first intercellular metabolite of extracellular D-glucose that caused the repression of Xyr1 target genes and *xyr1* [11]), D-xylose, or sophorose (induction of Xyr1 target genes) by CD spectroscopy measurements. Please note that the data with sophorose are not shown since they are highly similar in comparison to those observed with D-xylose. All three carbohydrates cause a change in the Xyr1 secondary structure, the extent of which directly correlates with the amount of carbohydrate present (Figure 4a,b). Notably, the greater loss of α-helix structures occurred in the presence of D-xylose or sophorose (up to 31%) compared to D-glucose-6-phosphate (21%) (also compare Figure 4a,b). This suggests that Xyr1 assumes different conformations depending on the carbohydrate used. Again, as a complementing experiment, EMSA analysis using the *xyn1* URR fragment as a probe and the same ratios of protein to carbohydrate as for the CD spectroscopy analyses was performed. Interestingly, the induced changes in secondary structure did not interfere with the ability to form protein–DNA complexes, since exactly the same shifts were obtained, regardless if carbohydrate was added and in which carbohydrate-to-protein ratio (data not shown).

The same assay was applied to Xyr1_A824V_ and analysed by CD spectroscopy. Interestingly, in contrast to the wild-type Xyr1, in this case, no different results were obtained for D-glucose-6-phosphate in comparison to D-xylose or sophorose (Figure 4c,d). Again, we performed BN-PAGE with both proteins and the carbohydrates as a control experiment to exclude that the observed differences are due to a changed protein–protein interaction (see Supplementary Data, Figure S1b). These findings are in good accordance with the carbon source-independent expression of (hemi)cellulases in the respective *T. reesei* mutant strain. Furthermore, it supports the assumption that the mutation is located in a domain that signals the carbohydrate presence. However, the observed changes in secondary structure at a high carbohydrate-to-protein ratio did not, again, affect the DNA–protein complex formation, according to EMSA analysis (data not shown), pointing to other regulatory mechanism(s) than mere DNA binding.

## 4. Discussion

As mentioned above, a mutant of an industrially used *T. reseei* strain showed an unusual deregulated and enhanced xylanase expression profile that prompted transcriptional analyses of prominent xylanase- and cellulase-encoding genes and their common transactivator-encoding gene, *xyr1* [10]. In the present study, we aimed to identify a mechanistic explanation for the observed phenotype by investigating the effect of the identified A824V mutation in Xyr1 [10] on the protein structure. While we could generally observe a different secondary structure when comparing the CD spectrum of the wild-type Xyr1 and the Xyr1_A824V_ consistent with a considerable loss of helical content in the mutant Xyr1, we also found a reduced capability of Xyr1_A824V_ for the protein–DNA complex formation. This finding was rather the opposite of what we expected because Xyr1 is the essential transactivator of the xylanase expression, and the mutation led to an increased xylanase expression. However, the lower DNA-binding affinity of Xyr1_A824V_ was supported by the comparison of the Kd of both the wild-type Xyr1 and the Xyr1_A824V_ for the corresponding part of the URR of *xyn1*. This observed reduced binding affinity in combination with the ability of the carbohydrates to induce a conformational change in the wild-type Xyr1, but not in the A824V mutant, suggests that there is more at play in the xylanase-deregulated phenotype of the mutant strain than just a loss of DNA-binding affinity. Of course, it remains open if the DNA-binding affinity plays a major role in regulating the expression of cellulase-encoding genes as they strictly follow the *xyr1* expression pattern, unlike the xylanase-encoding genes such as *xyn1*, the URR of which was used for investigations during this study. In contrast, a strong deregulation (i.e., a general loss of repression and induction) was reported for the xylanase expression. This makes the xylanase regulon an interesting research target and prompted us to investigate a possible allosteric response of Xyr1 in an NR-like way.

The modulation of the transactivating function of Zn_2_Cys_6_ binuclear cluster proteins in response to metabolic signals has been reported before. Wang and colleagues reported that the activating regulatory protein LEU3 of *Saccharomyces cerevisiae* [25,26] undergoes a conformational change in the presence of isopropylmalate, a metabolic intermediate of the biosynthesis of leucine, which exposes its transcriptional activation domain [27]. In the case of GAL4, the best-studied of the Zn_2_Cys_6_ binuclear cluster proteins, a masking of the transcriptional activation domain occurs through the regulator GAL80. In the presence of galactose, a third protein, GAL3, forms a heterodimer with GAL80, thus, freeing the GAL4 activation domain (reviewed by [28]). Besides a modulation of the activity of GAL4 through intermolecular interactions, there is evidence for a direct mechanism of inhibition by D-glucose. Stone and Sadowski showed that in the central region of GAL4, there is a domain responsive to D-glucose with an intramolecular role in inhibiting the transcriptional activity of the factor. In this model, the hypothesis of a direct interaction of D-glucose or its metabolites triggering the conformational change in GAL4 is considered [29]. Notably, this study provides one of the so far rare evidences of a direct interaction between a fungal transcription factor and a carbohydrate molecule. Until now, such findings were usually reported for prokaryotic regulatory systems (e.g., the *Escherichia coli* lac or gal operons). For eukaryotes, the study has the impact that more direct regulatory mechanisms should not be per se excluded.

In the case of the closely related orthologue of Xyr1 in *A. niger*, XlnR, the proposed model for repression by D-glucose (provided by [16]), was speculated to occur through an inter- or intramolecular interaction with the C-terminal region of the protein, which would result in an inactive state. It is noteworthy that Xyr1 has a high sequence identity in this region, including the V756 residue of XlnR, as described above. This amino acid correlates with position 821 in Xyr1, close to the described mutation, A824V.

**Figure 5:**
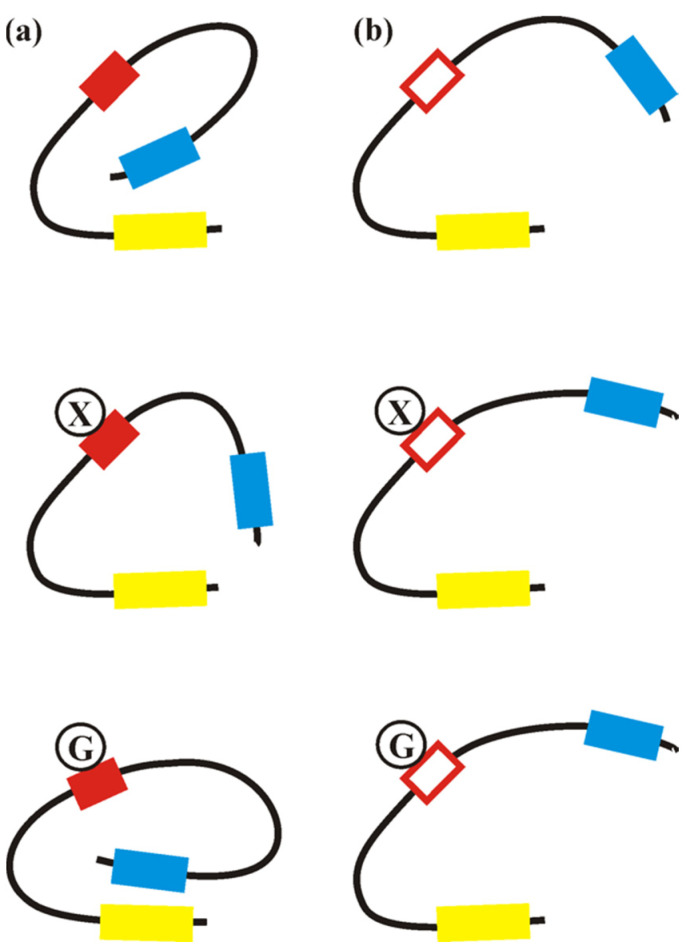
Schematic presentation of the allosteric response of Xyr1 variants. A simplified model of the structure of the wild-type Xyr1 (**a**) and the Xyr1_A824V_ (**b**) without carbohydrates and their conformational change in the presence of D-xylose (X) or D-glucose-6-phosphate (G), respectively, is presented. Yellow box, DNA-binding domain; red box, NR-like domain; red-framed box, NR-like domain bearing the A824V mutation; blue box, transactivating domain.

Notably, we found that the allosteric response of Xyr1 to three carbohydrates, which are responsible for either an induced or repressed gene expression in the parental strain, was lost in the mutant strain expressing Xyr1_A824V_. These observations strongly suggest that the C-terminal part of the Xyr1 protein functions as a NR-like domain, which is in good accordance with the observed deregulation of the xylanase expression. In addition, we found that in the wild-type Xyr1, the response to D-glucose-6-phosphate is different compared to inducing carbohydrates such as D-xylose or sophorose (compare Figure 4a,b). In our postulated model (see Figure 5), we propose that the mutation in the NR-like domain of Xyr1 leads to an altered presentation of the activation domain, independently of the carbohydrate signal (Figure 5b). In contrast, the intact NR-like domain in the wild-type Xyr1 would, in the simplest scenario, interact directly with the carbohydrate and expose or hide the transactivating domain according to the received signal (Figure 5a). To study this more extensively, a crystallographic study of Xyr1 variants alone and in the presence of carbohydrates would be beneficial.

Overall, this study supports the usefulness of engineering transcription factors as a beneficial tool to change their mode of regulation for desired properties, e.g., in this case, the carbohydrate-independence response. This is also well reflected by a number of studies, whereby a mutated Xyr1 was used for an improved enzyme expression in *T. reesei* [30,31,32], but also in other organisms such as *Penicillium oxalicum* [33].

## Figures and Tables

**Table 1:** Oligonucleotides used in the study.

Name	Sequence (5′–3′)	Employment
Pxyn1f_FAM	[FAM]- TTGGCAGGCTAAATGCGACATCTTAGCCGGATGCA	EMSA
Pxyn1f	TTGGCAGGCTAAATGCGACATCTTAGCCGGATGCA	CD
Pxyn1r	TGCATCCGGCTAAGATGTCGCATTTAGCCTGCCAA	EMSA/CD
CKT067	CACTCCACATGTTAAAGGCGCATTCAACCAGCTTC	EMSA
CKT068	GAAGCTGGTTGAATGCGCCTTTAACATGTGGAGTG	EMSA

## Data Availability

The data presented in this study are available in the main article and Supplementary Material.

## Supplementary Materials

The following supporting information can be downloaded at: https://www.mdpi.com/article/10.3390/jof8121254/s1, Figure S1. Blue native polyacrylamide gel electrophoresis of the wild-type Xyr1 and the mutant Xyr1_A824V_.
