# Supplementary material for: In Vitro Characterization of a Nuclear Receptor-like Domain of the Xylanase Regulator 1 from Trichoderma reesei
Source: J Fungi (Basel). 2022 Nov 27;8(12):1254. doi: 10.3390/jof8121254 (PMC9784857; doi:10.3390/jof8121254)
Supplement: Supplementary file 1 [file jof-08-01254-s001.zip › jof-2007978-supplementary.pdf]

Figure S1

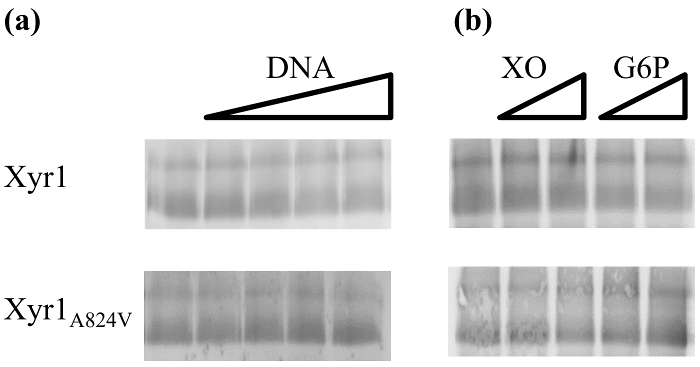

Blue native polyacrylamide gel electrophoresis of the wild-type Xyr1 and the mutant Xyr1$_{A824V}$. 200 nM of protein was applied together with **(a)** the *xyn1* URR fragment (-430 to -396 bp from ATG) (DNA) in molar ratios of 1:0, 5:1, 1:1, 1:5, and 1:10 or together with **(b)** D-xylose (XO) or D-glucose-6-phosphate (G6P) in molar ratios of 1:0, 1:1, and 1:5 (protein to carbohydrate).
